# Sleep disorders in patients with bipolar disorder: age and tobacco consumption correlates

**DOI:** 10.1192/j.eurpsy.2024.904

**Published:** 2024-08-27

**Authors:** K. Razki, C. Najar, S. Ben Aissa, M. Oumaya, R. Bouzid

**Affiliations:** ^1^Razi Hospital, LaManouba; ^2^Mental Health Department, Tahar Maamouri Hospital, Nabeul, Tunisia

## Abstract

**Introduction:**

Sleep disruptions are frequently observed in individuals with bipolar disorder and have been linked to various unfavorable consequences, such as an elevated risk of relapse and lower quality of life. Nonetheless, the impact of sociodemographic factors on the development and progression of these disruptions remains largely unexplored. Gaining insight into the relationship between sleep disruptions and sociodemographic factors is essential for designing effective interventions and enhancing clinical outcomes for individuals affected by bipolar disorder

**Objectives:**

The objective of this study is to examine the association between sleep disorders in patients with bipolar disorder II (BDII) and sociodemographic characteristics.

**Methods:**

This is a cross-sectional, descriptive, and analytical study that was conducted over a one-month period from October 1 to October 31, 2022, with patients attending the follow-up unit of the mental health department at Nabeul Hospital ,Tunisia.The study employed a questionnaire as a tool for data collection, and participants provided voluntary and informed consent before responding. The protection of participant confidentiality and anonymity was carefully observed during all stages of the study.

**Results:**

In this study, we enrolled patients who satisfied the following eligibility criteria: age range of 18 to 60 years, a confirmed diagnosis of type II bipolar disorder based on DSM V criteria, and psychiatric stability as demonstrated by no hospitalization within the preceding 6-month period.

Our study included a sample of 40 male patients diagnosed with type II bipolar disorder. The participants had a mean age of 36 ± 13.2 years, and the majority were unmarried and living with their families or alone. Over two-thirds of the participants had attained a university level of education, while a large proportion of the patients, specifically 80%, reported being regular smokers.

The results of the study revealed that the mean global score on the Pittsburgh Sleep Quality Index (PSQI) was 7.28 ± 3.35, indicating an overall low quality of sleep. The majority of the participants, that is 65% (26), had poor sleep quality scores (> 5), while 45% (18) reported experiencing poor sleep (PSQI ≥ 8).

Our analyses further demonstrated that there was a significant association between tobacco consumption and PSQI scores (p=0.003). Additionally, we found that participants who were above 40 years old had a higher likelihood of experiencing sleep disturbances (p=0.0017).

**Conclusions:**

According to the findings of our study, it appears that patients diagnosed with type II bipolar disorder may experience impaired sleep quality, which can be influenced by age and tobacco consumption. These results underscore the need for a holistic approach to patient care that addresses both the biological and sociodemographic factors that can impact sleep in this population.

**Disclosure of Interest:**

None Declared

